# Microbiological Analysis and Content of Heavy Metals in Different Candies, Chocolates, and Their Wrappers in Bangladesh

**DOI:** 10.1155/2024/4536475

**Published:** 2024-03-26

**Authors:** Kazi Milenur Rahman Prattay, Md. Tanvir Ahmed Chowdhury, Md. Raihan Sarkar, Tanvir Rahman, Sharmin Zaman Emon, Abrar Hossain, Hredoy Ghosh Rajib, Md. Abdurrahim

**Affiliations:** ^1^Department of Clinical Pharmacy and Pharmacology, Faculty of Pharmacy, University of Dhaka, Dhaka 1000, Bangladesh; ^2^School of Pharmacy, BRAC University, Dhaka 1212, Bangladesh; ^3^Department of Soil, Water and Environment, Faculty of Biological Sciences, University of Dhaka, Dhaka 1000, Bangladesh; ^4^Department of Pharmaceutical Technology, Faculty of Pharmacy, University of Dhaka, Dhaka 1000, Bangladesh; ^5^Centre for Advanced Research in Sciences (CARS), University of Dhaka, Dhaka 1000, Bangladesh; ^6^Department of Disaster Science and Climate Resilience, Faculty of Earth and Environmental Sciences, University of Dhaka, Dhaka 1000, Bangladesh; ^7^Biomedical and Toxicological Research Institute (BTRI), Bangladesh Council of Scientific and Industrial Research, Dhaka, Bangladesh

## Abstract

Present study investigates 39 brands of candies, chocolate, and litchis, purchased from Dhaka City, Bangladesh, for their moisture content, sulphated ash value, heavy metal, and bacterial contamination. All the brands showed moisture content (0.64%-4.775%) within the BSTI range, but sulphated ash values (18.80%-25.72%) were beyond the accepted value. Pb, Cd, Ni, and Cr ranged from 0.24-2.40 *μ*g/g, 0.071-0.44 *μ*g/g, 0.38-48.10 *μ*g/g, and 0.50-12.79 *μ*g/g, respectively, in the tested brands. Most of the brands contained Pb and Cd beyond the acceptable limits of WHO/FDA. Pb (2.24-2586.75 *μ*g/g) was found in high concentration in the packaging of most brands, and Ni and Cd ranged from 2.10-108.05 *μ*g/g and 1.68-45 *μ*g/g, respectively. Bacterial presence was found in 15 brands, and 4 of them had a total aerobic bacterial count of >1 log CFU/g. Consumption of such contaminated confectionaries holds significant public health risks, specially in children, and demands necessary precautionary steps.

## 1. Introduction

The status of the well-being of young generation, especially children, determines the public health scenario as well as the overall prosperity of a nation in the near future [1]. When it comes to any form of food chain contamination, children constitute the most vulnerable age group since very often, they intake more food per body weight than the adults causing greater exposure to the contaminants, leading to acute and/or long-term hazardous impact to health [2, 3]. Therefore, food quality and safety must be carefully assessed and regulated, especially those that are mainly consumed by young children.

Confectionary such as candies (lozenge and toffee) and chocolates are widely consumed by, but not restricted to, children all over the world, especially in the developing countries [4, 5]. These are popular among children due to their affordability as well as sweetness, flavors, colors, shapes, etc. [5, 6]. Most of the ingredients in candies, such as sugar, water, chocolate, honey, milk, and sweeteners (e.g., aspartame), are derived from plant or animal sources [6]. Liquid glucose, soya solids, vegetable oil, malt extract, emulsifiers, buffering agents, sodium bicarbonate, salts, wheat flour, flour improvers, yeast, flavors, etc. are additional materials used in the production of candies and chocolates. As the number of different components used in making candies/chocolate is growing, the complexity of the finished goods is rising, ultimately leading to higher chances of contamination of the finished goods, especially heavy metals, and microbial and particulate contamination [7].

Heavy metal contamination of candy/chocolates can happen at any stage of the production process, including raw materials, processing, packaging, transportation, storage, and marketing, all the way through to consumption [5, 6]. Milk solids, cocoa, hydrogenated vegetable oils, permitted emulsifiers, buffering agents, etc. used as raw ingredients in candy or chocolates often act as possible sources of nickel (Ni), cadmium (Cd), and lead (Pb) [1]. Metals like Pb, Cd, Chromium (Cr), etc. are often introduced to the candies when their surface is sticky and allow the candy surface to cling to the inner cover of the package [5–8]. Trace elements may also migrate through the printing ink from the packaging material into the candy surface due to poor design [2, 9]. Nickel is introduced in the final product as it is used as a catalyst for the hydrogenation of unsaturated fatty acids for the hardening of chocolates [1]. Studies conducted in different countries, for instances, India, Nigeria, Saudi Arabia, and Turkey, reported a high amount of toxic heavy metals in chocolates and candies available in their country [7, 10–12]. According to BDS, the maximum acceptable limit of Ni in toffees is 1 mg/kg [13]. Ingestion at a higher level might cause nausea, dizziness, weakness, bronchial hemorrhage, etc. [1]. The maximum acceptable limit of cadmium in chocolate is 0.1 *μ*g/g according to the US FDA, and excess ingestion might result in gastrointestinal pain, nausea, diarrhea, respiratory distress, renal damage, hypertension, impaired reproductivity, etc. [1, 6]. Lead is a classic toxic metal and taken over the maximum acceptable dose (0.1 *μ*g/g in chocolates according to both US FDA and FAO/WHO and 1 *μ*g/g for BDS) and can demonstrate renal, cardiovascular, hematopoietic, immune, and reproductive system toxicity which may even lead to death if Pb is taken in a very high dose [2, 6, 14]. Chromium can produce renal toxicity, hypersensitivity, and carcinogenic effect [15].

Candies and chocolates are often poorly packaged and stored allowing microbial contamination, and absence of any preconsumption treatment allows the total microbial load to get introduced into the body [4]. Children are usually more susceptible to food-borne pathogens since organs like stomach and intestine as well as the immune systems are still under development, and therefore, ingestion of a smaller quantity of microbial load often produces a more detrimental effect in children compared to their adult counterparts [4, 16, 17]. Coliforms, *Escherichia coli*, *Salmonella* spp., *Pseudomonas* spp., and *Bacillus* spp. are some commonly found microorganisms found in foods and can cause adverse clinical symptoms in the human body [4, 18–20]. According to the International Commission on Microbiological Specification of Foods (ICMSF), the acceptable limit of aerobic mesophilic bacterial count in confectionaries like chocolates and candies should not overrun a maximum of 10^3^ CFU/g [21].

Moisture content is a widely monitored parameter in the quality control of food items, specially for chocolates and candies. Total moisture content can affect the texture of the finished goods and thereby consumer's compliance. Low moisture content can make the finished product brittle, but excessive moisture can confer it sticky and unpleasant [22]. Inappropriate packaging and poor storage condition can often lead to high moisture content in chocolate and candies as they contain a high content of sugar and therefore may favor the survival and/or growth of microorganisms in the product [23]. According to BDS, the required moisture content in lozenges is 3% (w/w). In addition, sulphated ash value is often determined to evaluate the amount of inorganic contaminants in chocolates and candies, and the required value is 3% (w/w) according to BDS [14].

There are many local and international brands of candies and chocolates across the Bangladeshi markets, and to the best of our knowledge, no study has been conducted to assess and monitor the quality and safety of these food items as well as their wrappers in this country. In addition, few studies are reported across the world to assess the microbial contamination in candies/chocolate [4].

The aim of the current study was to assess the quality of some local manufacturing candies and chocolates as well as the standard of imported products available in the Bangladesh Market, in terms of their moisture content, sulphated ash value, and microbial and heavy metal contamination. The presence of toxic heavy metal contents in the packaging material of these candies and chocolates was also investigated. The findings of the study will be supportive to spread awareness among mass population as well as concern the associated regulatory authorities.

## 2. Materials and Methods

### 2.1. Materials

Chemicals such as hydrogen peroxide, acetonitrile, hydrochloric acid (HCl), sulphuric acid (H_2_SO_4_), nitric acid (HNO_3_), sodium chloride, disodium hydrogen phosphate, potassium hydroxide, potassium dihydrogen phosphate, and different types of agar media were bought from local vendors. Spatula, Petri dishes, glassware, filter paper, aluminium foil, dropper, syringe, cotton, bottles, and glass pipettes were procured from local vendors.

### 2.2. Sample Collection

A total of 39 brands, both locally manufactured and imported, of chocolates, candies/lozenges, and litchis were purchased (30-100 samples of each brand) from local shops (various super shops, department stores, grocery stores, and retail shops) at different locations of Dhaka City, Bangladesh. Among the 39 brands, the number of locally produced brands was 27, 4 were from imported brands, and 8 were from locally manufactured international brands. The number of brands selected for candies, chocolates, and litchis were 27 (hard candy: 22; toffee: 5), 9, and 3, respectively. Although 25 individual samples were used for the investigation of each chocolate and candy brand, only 10 individual samples were used for each brand of litchis since the litchis were only assessed for the microbial contamination (Table 1). All these products were available in the local markets and valid for human consumption. The analysis of the samples was performed before the expiry date. The sample names were blinded and given codes (Table 1). All the collected chocolates, candies, and litchis were stored in a dark and dry place at room temperature. Each sample was analyzed three times, and other samples were kept under sealed condition for future as evidence.

### 2.3. Moisture Content Analysis

The moisture content of the samples was measured by Halogen Moisture Analyzer machine (Model XY105MW, China) following the procedures used by Elbl et al. [24], with some alterations. Chocolates/candies were taken out from the package and triturated quickly. The machine was started and kept that way for about 30 minutes before introducing the sample. 2 g of accurately weighed sample was taken in the tray of the analyzer, and the measurement was done using thermogravimetric principle. The sample was heated until a constant moisture content value was achieved. Moisture content analysis was performed three times for each brand of chocolate and candy.

### 2.4. Sulphated Ash Determination

Sulphated ash values for the samples were determined by following the standard protocols described by [25]. Briefly, 2 g of chocolate/candy samples were taken and crushed into powder using a clean pestle and mortar. Concentrated sulphuric acid (1.5 ml) was added to the sample, and the sample was carbonized by heating gently. Muffle furnace was used to convert carbonized material to ash at 600°C. The ash was allowed to cool. Then, the samples were heated again with concentrated sulphuric acid using a hot plate and kept until the sulphuric acid fume was stopped. Then, the ash samples were again heated in muffle furnace for 2 h. The weight of the raw and ash samples was recorded. The same process was repeated in muffle furnace for about 30 min, and the lowest weight of the sample was recorded. Finally, the sulphated ash values of the samples were determined by using the following equation.

(1) $$\begin{array}{c} \text{Sulphated ash}\left(\%\right)=M_{1}\times \frac{100}{M_{2}}, \end{array}$$

where *M*_1_ is the mass of ash (g) and *M*_2_ is the mass of sample (g).

The sulphated ash analysis was conducted three times for each of the brands of chocolates and candies.

### 2.5. Heavy Metal Measurement

#### 2.5.1. Sample Processing and Preparation for Analysis

The chocolate and candy samples were triturated and dried in the oven at 70°C for 1 h. For each of the samples, about 2 g of packaging material was taken and was burned at 650°C for 6.5 h in the muffle furnace, and the resultant ash was collected and weighed. The ash of the packaging materials and candy/chocolate samples was digested using a block digester. The prepared ash of the packaging materials and 1 g of ground and dried candy/chocolate samples were then put into a glass digest tube, concentrated nitric acid (5 ml) was added to the tube, and predigestion took place overnight. Just before digesting, 1 ml of hydrogen peroxide was added to the sample, and the samples were then heated on the block digester for 0.5 h at 60°C, for 1 h at 80°C, for 1.5 h at 100°C, and finally, at 120°C for 0.5 h until the solution became clear. The digested samples were allowed to cool down and then transferred into 25 ml polypropylene tubes, and finally, the volumes were made up to 25 ml mark.

#### 2.5.2. Chemical Analysis

The packaging materials and chocolate/candy samples were analyzed for total cadmium, chromium, lead, and nickel using AAS (Varian AA-240 and PinAAcle 900T AA Spectrometer). The quality control/quality assurance of the analysis was maintained following the standard procedure.

#### 2.5.3. Heavy Metal Analysis Method Precision and Accuracy

The analysis method of heavy metals was calibrated by taking three known concentrations of each metal and measuring the absorbance three times each. Percentage recovery was 101.03%, 101.88%, 102.34%, and 101.1% for Pb, Cr, Ni, and Cd, respectively. The calibration data is mentioned in Table 2.

### 2.6. Microbiological Assay

#### 2.6.1. Preliminary Microbiological Screening

The spread plate technique and drop plate method were used to preliminary screen the presence of bacteria in all 39 brands [26].

*(1) Agar Plate Preparation*. In a conical flask, 30 g of nutrient agar that was dissolved in 1 liter of distilled water was added. The prepared agar solution and enough plates were sterilized in the autoclave machine for 15 minutes with 121°C temperature and 15 PSI pressure. The sterilized plate is set up in the laminar flow cabinet, and the hot solution of agar of around 15-20 ml is poured into each of the sterilized plates. The plates are kept in the cabinet till the agar solution is solidified in the plate. Then, the plates are transferred into the incubator machine at 37.5°C for 24 hours to check the contamination [26, 27].

*(2) Chocolates and Candy Sample Solution Preparation*. A concentration of 0.85% (w/v) saline solution was prepared in the conical flask with appropriate measurement of sodium chloride (NaCl) and sterilized in the autoclave with the same protocol as above. The saline solution was then transferred to a stomacher bag with the measurement, for 10 g of candy and 90 ml of saline solution, as mentioned. Candies were then dissolved in the stomacher bag and kept into the homogenizer machine for 2 minutes for proper dissolving of the candies into the saline solution. After that, all the stomacher bag with the candy solution was transferred to the laminar flow cabinet. The agar plate that was prepared earlier was checked for contamination, and the plate with no contamination was selected for checking the bacterial growth in the candy solution.

*(3) Spreading Plate Technique*. 100 *μ*l of candy solution from each of the stomacher bags was placed drop by drop into the agar plates, respectively, by using micropipette. Candy solution was then spread with a glass spreader into the agar plate until the solution is fully dissolved into the agar plate. The agar plates were finally kept in the incubator machine invertedly at 37.5°C for 24 hours to check the growth of bacteria [26, 28].

*(4) Drop Plate Method*. From each of the stomacher bags containing the candy solution, 1 ml of solution was added to the 9 ml of saline solution in the falcon tube for 10 times dilution. Then, from the falcon tube, 10 *μ*l of the candy solution was placed drop by drop in the agar plate. The agar plates were finally kept in the incubator machine at 37.5°C for 24 hours to check the growth of bacteria [29].

#### 2.6.2. Specific Bacteria Identification and Bacterial Count

Among 39 samples, the presence of bacteria was detected in 15 samples. These samples were further investigated for bacterial count and to identify specific bacteria.

*(1) Selection of Media*. For specific count of bacteria, different types of media were selected. Different specific bacteria and selected respective media are enlisted in Table 3.

*(2) Preparation of Media*. In separate conical flasks, for specific growth of bacteria, specific bacterial media were selected and dissolved in water as labelled. For 1 liter of solution, the weight of media taken is mentioned in Table 3.

*(3) Sterilization*. The prepared media except xylose lysine deoxycholate agar and enough plates were sterilized in the autoclave machine for 1 hour with 121°C temperature and 103 kPa pressure. Xylose lysine deoxycholate agar was sterilized in the microwave oven at 100°C. The sterilized plate was set up in the laminar flow cabinet, and the hot solution of bacterial media of around 15-20 ml was poured into each of the sterilized plates. The plates were kept in the cabinet till the media solution was solidified in the plate. Then, the plates were transferred into the incubator machine at 37.5°C for 24 hours to check the contamination [26].

*(4) Enrichment*. Tryptic Soy Broth (TSB) media of around 50 ml was added into each of the stomacher bags containing the candy solution. TSB media was used for the enrichment and cultivation of bacteria that were not excessively fastidious. The stomacher bag was then preserved for 24 hours.

*(5) Streaking Method*. Streaking was done by bacterial inoculation loop. Using the flame, the inoculation loop was first sterilized. After cooling the loop, it was dipped into the stomacher bag containing the sample and TSB. The loop was then dragged across the surface of bacterial media from side to side in a crisscross motion till nearly 30% of the plate had been covered. Then, the media plates were kept in the incubator machine at 37.5°C for 24 hours to check the growth of bacteria.

### 2.7. Statistical Analysis

All the statistical analysis was performed using IBM SPSS Statistics 25.0. All the numerical data were checked for their normality and were transformed accordingly, if necessary, prior to all parametric tests. One-way ANOVA test was performed to investigate the significance of the association between categorical and continuous variables. *P* value of less than 0.05 was considered significant for all statistical analyses.

## 3. Results and Discussion

### 3.1. Moisture Content (%) Analysis

Water is a common constituent of chocolate and candies. The amount of moisture effects the product's overall quality, stability, texture, and other physical and chemical properties [22]. Final moisture content is directly related to the overall stability of the product as more water presence in the sample and the chance of microorganism contamination will also be increased. According to the BSTI, the maximum acceptable limit of moisture in toffees is 8%, and for lozenges, it is a maximum of 3% [13, 25]. A total of 22 brands of hard candies were assessed for moisture content, and the range was found 0.64 to 4.775%. High moisture was found in samples S3 (4.775%), S6 (4.335%), and S13 (4.04%), and low amount of moisture was detected in S20 (0.640%), S19 (0.70%), and S11 (0.945%). Among the 5 toffee brands investigated, S5 (3.34%) had the highest moisture content whereas S8 (1.96) had the lowest moisture content value (Table 4). A previous study found hard candies and toffees to have moisture content between 2 to 5% and 6 to 18%, respectively, which is higher than what was found in the current study [30]. Moisture content analysis revealed that not all the candies contained moisture within the standard range set by BSTI and other international regulatory agencies. Different brand chocolates were also assessed and found the moisture contents within the range of BSTI (0.880 to 1.96%; Table 4).

### 3.2. Sulphated Ash Analysis

This analysis was carried out to determine the content of inorganic impurities in the chocolates and candies [31]. Bangladesh standards and testing institution sets the maximum limit of sulphated ash at 3% and 2.5% for lozenges and toffee, respectively [13, 25]. The range of sulphated ash in tested candies was 18.80% to 25.72% which indicated the presence of high amount of inorganic residue compounds (Table 5). Sulphated ash analysis of chocolates revealed that they also contained a larger amount of inorganic residue (Table 5).

### 3.3. Heavy Metal Analysis in Candies and Chocolates

Heavy metal content in candies and chocolates has been analyzed. The presence of lead (Pb), chromium (Cr), nickel (Ni), and cadmium (Cd) in different brands of chocolates and candies was assessed. According to BSTI, the maximum allowable concentration of Pb, Cd, and Ni in lozenge and toffee is 2 *μ*g/g, 1 *μ*g/g, and 1 *μ*g/g, respectively [13, 25]. However, the US FDA and WHO/FAO set the maximum permissible limit for Pb in chocolates as 0.1 *μ*g/g, and for Cd, it is 0.1 *μ*g/g (USFDA) and 0.05 *μ*g/g (FAO/WHO) [7]. Levels of cadmium in chocolates for Germany (0.4 *μ*g/g), Finland (0.5 *μ*g/g), Poland (0.05 *μ*g/g), and Malaysia (1 *μ*g/g) have been set as the maximum allowable limit [32].

Among 27 brands of analyzed candies, most of the samples contained a greater level of Pb than the maximum permissible limit set by FAO/WHO/FDA. The levels of Pb in all candy samples in which Pb has been detected ranged from 0.24 *μ*g/g to 2.40 *μ*g/g (Table 6). For instance, S10, S1, S8, S6, S11, S2, S9, S12, S13, S15, S16, S17, S18, S19, and S20 candies have more than 1 *μ*g/g Pb (Table 6). The highest Pb concentration was observed for sample S10 (2.40 *μ*g/g). Candies S32, S33, S34, and S35 have no or below detection limit Pb. The Pb was detected in S14 (1.176 *μ*g/g), S31 (1.015 *μ*g/g), S27 (0.912 *μ*g/g), S28 (0.859 *μ*g/g), and S30 (0.908 *μ*g/g). The range of Pb in chocolates was found at 0.86 to 1.18 *μ*g/g. Pb was not detected or might be below the detection limit in four chocolate samples—S35, S36, S37, and S39. However, the level of Pb in most (except S10) of the brand chocolates and candies was lower than the standard limit set by BSTI [14]. Dahiya et al. reported a level of 0.049 to 8.04 *μ*g/g of Pb in 69 different brands of chocolates in India [1]. Villa et al. found a concentration range of 21–138.4 ng/g of Pb in 30 chocolate samples in Brazil [33]. Concertation of Pb was detected at 1.11-2.48 *μ*g/g in various chocolates and candy samples of Pakistan [32]. A high amount of Pb exposure could cause a negative impact on cardiovascular, neurological, renal, and hepatic functions [34]. The impact is more serious for children and causes problems related to behavioral and cognitive disorders as well as impairs neurodevelopment [35]. For this, USFDA allowed a maximum level of Pb 0.1 mg/kg (0.1 *μ*g/g) in both chocolates and candies [34, 36]. If the USFDA guideline is considered, then almost all the chocolate and candy samples tested in the current study exceed the maximum allowable limit of Pb (0.1 *μ*g/g).

Many studies reported that dietary exposure of high amount of Cd causes kidney and liver damage [37–39]. The level of Cd ranging 0.071–0.44 *μ*g/g in tested candy samples was below the set standard of 1 *μ*g/g by BSTI [25]. However, if the USFDA (0.1 *μ*g/g) and FAO/WHO (0.05 *μ*g/g) maximum tolerability limit for Cd is considered standard, then most of the tested candy samples contained a high amount of Cd [7]. The concentration range of Cd for chocolate samples was 0.17 to 0.43 *μ*g/g which was also beyond the USFDA and WHO/FAO maximum tolerability limit. Jalbani et al. found the Cd range 0.099-0.353 *μ*g/g in 40 different brands of candies and chocolate in Pakistan [32]. The mean level of Cd has been reported as0.17 ± 0.22 *μ*g/g in some chocolates and candies collected from grocery shops in Hisar District, India [7]. Dahiya et al. found Cd concentration range from 0.001 to 2.73 *μ*g/g in chocolate products of India [1].

The exposure of high amount of nickel may increase the possibility of cancer and dermatitis as well as lung and kidney impairment, bronchial hemorrhage, dizziness, etc. [1, 40]. Intake of Ni more than 8 *μ*g/Kg per day may elevate the chance of skin eczema [40]. The maximum permissibility limit of Ni is 1 *μ*g/g in candy product by BSTI [13]. The Joint FAO/WHO Expert Committee sets a daily tolerable intake of Ni not more than 5 mg/kg/d [11]. The range of nickel concentration in different candy samples was 0.38 to 48.10 *μ*g/g (Table 6). On the other hand, for chocolates, the range was observed at 0.83 to 1.57 *μ*g/g. Among 36 samples, S1 (13.24 *μ*g/g), S2 (1.48 *μ*g/g), S3 (3.1 *μ*g/g), S6 (1.43 *μ*g/g), S8 (48.10 *μ*g/g), S10 (14.78 *μ*g/g), S11 (1.28 *μ*g/g), S16 (1.19 *μ*g/g), S18 (2.89 *μ*g/g), S19 (1.19 *μ*g/g), S23 (1.25 *μ*g/g), S27 (1.57 *μ*g/g), S28 (1.14 *μ*g/g), S30 (1.43 *μ*g/g), S31 (1.09 *μ*g/g), S35 (1.11 *μ*g/g), and S36 (1.32 *μ*g/g) showed high amount of Ni content. Selavpathy and Saraladevi and Dahiya et al. reported a range of 0.15–3.55 *μ*g/g and 0.041 to 8.29 *μ*g/g Ni, respectively, in different candies and chocolates of India [1, 41]. 1.45-4.33 *μ*g/g range of Ni was observed in various candies and chocolates in Pakistan [32]. The elevated amount of Ni detected in tested samples may be due to the use of cocoa butter and hydrogenated vegetable oil/trans-fat or may be leaching from the wrappers which have high Ni levels [1, 9, 42]. It is necessary for further investigation to confirm the exact reason for the high concentration of Ni.

Although a low amount of chromium is essential for the insulin activity, metabolism of protein, fat, and glucose, however, too much exposure of chromium (Cr) is toxic and causes detrimental health effects such as lung cancer, bronchial carcinoma, and gastrointestinal cancer [9, 11, 43]. The maximum dietary intake of Cr is 1.86 *μ*g/kg per day, and the Expert Group on Vitamins and Minerals (EVM) recommended that a maximum tolerable value of Cr (III) is 2.14 *μ*g/kg per day [11]. FAO/WHO set that the standard limit of Cr is 0.5 *μ*g/g in food additives [6]. Cr that had not been identified in most of the chocolates and candies might be due to low level or not presence (Table 6). Cr was detected in candies S32, S33, S34, and S38 and chocolates S31, S35, S36, S37, and S39. The range of Cr concentration is 10.73 *μ*g/g to 12.35 *μ*g/g and 0.50 *μ*g/g to 12.79 *μ*g/g in candies and chocolates, respectively (Table 6). These values were much higher than the reported value by Aychiluhimew. Aychiluhimew reported the Cr concentration range of 0.148 *μ*g/g to 0.208 *μ*g/g in chocolates and 0.17 to 0.181 *μ*g/g in candies [44]. Another study conducted in Nigeria on candy samples found Cr concentrations 0.001–3.093 *μ*g/g [6].

### 3.4. Heavy Metal Analysis in Packaging Materials

Plastic package or wrapper of chocolates and candies protect the product from outside environment. Most chocolate and candy manufacturers usually use very colorful wrappers; this might be due to tempt children to purchase the chocolates and candies [9]. Different metal-based inorganic pigments are used to print the level on outer surface of the package [9]. There are various possible causes of exposure of toxic heavy metals in chocolate and candy products [45]. Those migrations of heavy metals from wrappers to candy items are common as they closely adhere to the candy [45]. The quality of packaging material needs to be controlled properly. EU Directive (94/62/EC) sets a maximum limit as the sum of Pb, Cd, Cr, and Hg or their compounds in wrappers was 100 *μ*g/g in total [46]. Many studies reported that the presence of high amount of Pb, Cd, Ni, Cr, etc. contaminants in wrapper can migrate into the food product [9, 47]. The colors of most of the wrappers of the current study were green, red, blue, and yellow. Pb concentration range was found at 2.24–2586.75 *μ*g/g in the 33 wrappers of 36 tested candy and chocolates (Table 7). P36, P37, and P39 wrappers showed below detection level of Pb. A high amount of Pb has been observed in most of the packages of tested products; for instance, P30 (2586.75 *μ*g/g), P17 (988.01 *μ*g/g), P16 (761.94 *μ*g/g), P27 (687.5 *μ*g/g), P19 (637.27 *μ*g/g), P18 (581.76 *μ*g/g), etc. contained elevated level of Pb. Kim et al. detected 110.3–6394.1 *μ*g/g and 136.9 *μ*g/g-1429.3 *μ*g/g concentrations of Pb and Cr, respectively, in the candy packages [9]. Most of the wrappers contained Cr below the detection limit of the instrument. However, the level of Cr was found to be high in P32 (1763.75 *μ*g/g), P33 (1062.5 *μ*g/g), P38 (1112.45 *μ*g/g), P39 (1092.5 *μ*g/g), P34 (450.83 *μ*g/g), P37 (328.33 *μ*g/g), P36 (280.04 *μ*g/g), and P31 (2.49 *μ*g/g). Current study findings of Pb and Cr agreed with Kim et al.'s reported findings [9]. Ni was detected in all the tested chocolate and candy wrappers except P37 (Table 7). Tested wrappers of all 36 brands contained Cd. The concertation range of Ni and Cd was detected at 2.10-108.05 *μ*g/g and 1.68-45 *μ*g/g, respectively, in the package materials (Table 7). Dias and Wickramasinghe detected and reported Ni, Cr, and Pb concentrations at 2 to 30 *μ*g/g, 2 to 300 *μ*g/g, and 0.5 to 6 *μ*g/g, respectively, in different candy packages; however, Cd was not detected in their tested wrappers [42]. Even though all the heavy metals listed in the EU Directive were not analyzed in the current study, P3, P11-P23, P27, P28, P30-P34, P36, and P37-P39 contained heavy metals Pb, Cr, Ni, and Cd above the maximum tolerable limit at 100 *μ*g/g in total set by EU (94/62/EC) (Table 7) [46]. So, further study regarding the migration of heavy metals from wrappers into chocolate and candy products is highly desirable.

### 3.5. Heavy Metal Analysis for Local, Imported, and Locally Produced International Brands

**Figure 1:**
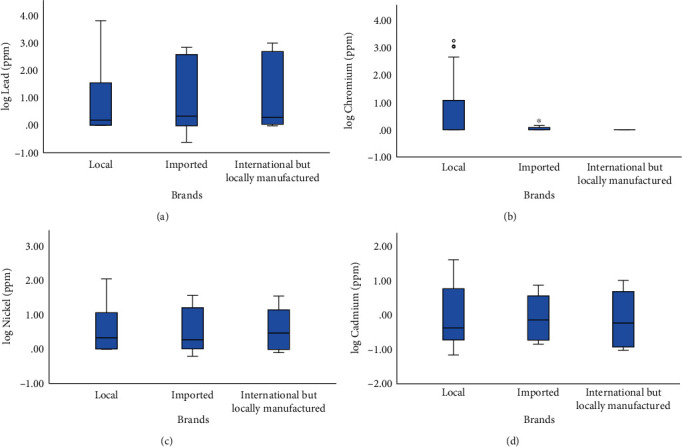
Box plots showing the association of type of chocolate or candy brands (local, imported, or locally manufactured international brands) with (a) log of lead concentration (*μ*g/g), (b) log of chromium concentration (*μ*g/g), (c) log of nickel concentration (*μ*g/g), and (d) log of cadmium concentration (*μ*g/g). ∗: extreme outlier value (more than 3.0 times the interquartile range away from the box); o: mild outlier value (more than 1.5 times the interquartile range away from the box).

One-way ANOVA indicated that the concentration of chromium found in the chocolates, candies, and their wrappings was significantly associated with the source of the brand (*F* = 4.077, df = 2, *p* = 0.021) where the samples from the local brands had a higher average concentration of chromium than the samples of imported and locally manufactured international brands (Figure 1). However, no such significant association was found for cadmium, nickel, and lead.

### 3.6. Microbiological Assay

Usually, candies and chocolates should be free from all types of microorganisms due to very low water activity to assist the growth of bacteria. However, the presence of Salmonella and *E. coli* in different chocolate and chocolate products has been reported in the UK, USA, Canada, Finland, and Norway [48].

Among 39 candy and chocolate brands, the presence of bacteria was confirmed in fifteen samples by initial screening. These fifteen different types of candy and chocolate samples were assessed for total aerobic bacterial count, total coliform count, *Escherichia coli*, *Salmonella* spp., *Pseudomonas* spp., and *Bacillus* spp.

The study revealed that samples S3, S4, S35, and S37 contain 3.48, 3.34, 3.41, and 3.49 log CFU/g of total aerobic bacteria, respectively (Table 8). Sample S3 showed a significant number of *Bacillus* spp. presence (2.6 log CFU/g), which indicated the poor quality of the sample. Samples 16, 26 (litchi), 32, 33, 35, 38, and 39 also exhibited the presence of stressed *Bacillus* spp. after enrichment. The major cause of chocolate and candy contamination might be due to not practicing GMP properly, contaminated raw material, high moisture content, substandard packaging, poor storage condition, etc. [49]. Other microbiological organisms tested, i.e., total coliform count, *Escherichia coli*, *Salmonella* spp., and *Pseudomonas* spp., were absent in all the samples even after enrichment. So, further study will be to check the presence of any toxin compound produced by *Bacillus* spp. and whether it is a concern for human health.

One of the limitations of the current study is the number of samples. In the future, more samples should be evaluated to find out the real quality picture of locally produced and imported brands of chocolates, candies, and litchis. Heavy metals like Hg, Se, and Sb and other toxic metals also need to be assessed. The migration study of heavy metals from packages to the product is also necessary to be performed. The presence of any toxin compound produced by *Bacillus* spp. and other microorganisms needs to be also evaluated. To evaluate the exposure and related health risks due to consumption of some of these heavy metals present in candies, litchis, and chocolates, daily intake and target hazard quotient will also need to be calculated. Moreover, carcinogenic and noncarcinogenic effects due to the intake of heavy metals present in these food items will also need to be predicted.

## 4. Conclusions

There are many locally produced and imported brands of chocolates, candies, and litchis that are available in the Bangladesh market. Among those, 39 brands of candy and chocolate were assessed for potential toxic metals and microbial contamination as well as quality in terms of moisture content and sulphated ash. Many of the tested products contained a high amount of heavy metals, and the concentration range found Pb (0.24 to 2.40 *μ*g/g), Cd (0.071-0.44 *μ*g/g), Ni (0.38-48.10 *μ*g/g), and Cr (0.5-12.79 *μ*g/g). Very colorful packages of candy and chocolate products were also evaluated for toxic heavy metals. Most of the wrappers contained a greater amount of Pb (range 2.24–2586.75 *μ*g/g), Cd (1.68-45 *μ*g/g), Ni (range 2.10-108.05 *μ*g/g), and Cr (range 2.49-1763.75 *μ*g/g), and few of them exceed the maximum limit 100 *μ*g/g in total, as set by EU Directive (94/62/EC). Microbial contamination has been assessed for all samples, and the presence of *Bacillus* spp. was detected in some candy and chocolate products. The presence of these heavy metals and microorganisms in the products could cause serious health hazards, specially for children who consume these items most frequently. Findings of this study will provide the responsible regulatory authorities with a current picture of the quality of different locally produced and imported brands of chocolates, candies, and litchis and inspire them to monitor the overall quality on a regular basis.

## Figures and Tables

**Table 1:** Given codes of tested chocolates, candies, and litchis.

Brand code	Local (L)/imported (I)/locally manufactured international brand (IL)	Candy (C)/chocolate (Ch)/litchi (Li)	Number of samples analyzed	Brand code	Local (L)/imported (I)/locally manufactured international brand (IL)	Candy (C)/chocolate (Ch)/litchi (Li)	Number of samples analyzed
S1	L	C (h)	25	S21	IL	C (h)	25
S2	L	C (h)	25	S22	IL	C (h)	25
S3	L	C (h)	25	S23	IL	C (h)	25
S4	L	C (t)	25	S24	L	Li	09
S5	I	C (t)	25	S25	L	Li	09
S6	L	C (h)	25	S26	L	Li	09
S7	L	C (t)	25	S27	I	Ch	25
S8	L	C (t)	25	S28	L	Ch	25
S9	IL	C (t)	25	S29	L	C (h)	25
S10	L	C (h)	25	S30	L	Ch	25
S11	IL	C (h)	25	S31	I	Ch	25
S12	L	C (h)	25	S32	L	C (h)	25
S13	IL	C (h)	25	S33	L	C (h)	25
S14	L	Ch	25	S34	L	C (h)	25
S15	I	C (h)	25	S35	L	Ch	25
S16	L	C (h)	25	S36	L	Ch	25
S17	IL	C (h)	25	S37	L	Ch	25
S18	IL	C (h)	25	S38	L	C (h)	25
S19	L	C (h)	25	S39	L	Ch	25
S20	L	C (h)	25				

(h) = hard candy, (t) = toffee.

**Table 2:** Calibration data for lead (Pb), chromium (Cr), nickel (Ni), and cadmium (Cd).

Metal	ID	Mean signal (Abs)	Entered conc. (mg/l)	Mean calculated conc. (mg/l)	Std dev	Average recovery (%)
Pb^a^	Blank	0.000	0	0.000	—	101.03
	A1	0.0227	1.0	1.095	0.0040	
	A3	0.0631	3.0	3.049	0.0002	
	A5	0.1024	5.0	4.949	0.0096	

Cr^b^	Blank	0.000	0	0.000	—	101.88
	B1	0.0235	1.0	1.048	0.0003	
	B3	0.0667	3.0	3.077	0.0059	
	B5	0.1053	5.0	5.007	0.0017	

Ni^c^	Blank	0.000	0	0.000	—	102.34
	C1	0.0241	1.0	1.071	0.0087	
	C3	0.0620	3.0	3.078	0.0015	
	C5	0.1009	5.0	4.982	0.0022	

Cd^d^	Blank	0.000	0	0.000	—	101.76
	D1	0.0214	1.0	1.037	0.0009	
	D3	0.0609	3.0	3.091	0.0041	
	D5	0.1077	5.0	5.015	0.0056	

^a^Correlation coefficient: 0.9994; slope: 0.0204; intercept: 0.0012. ^b^Correlation coefficient: 0.9986; slope: 0.0210; intercept: 0.0016. ^c^Correlation coefficient: 0.9984; slope: 0.0199; intercept: 0.0019. ^d^Correlation coefficient: 0.9986; slope: 0.0214; intercept: -0.0006.

**Table 3:** Selected types of media for different specific bacteria and amount of different media dissolved to make a 1-liter solution of the culture media.

Bacteria	Type of media	Amount (g/l)
Total aerobic bacterial count	Nutrient agar	28
Total coliform count	Sorbitol MacConkey agar	50
*Escherichia coli*	Tryptone bile glucuronic agar	39.6
*Salmonella* spp.	Xylose lysine deoxycholate agar	56.68
*Pseudomonas* spp.	Cetrimide agar base	46
*Bacillus* spp.	NGKG agar	26.5
Enrichment broth	Tryptic soy broth	31

**Table 4:** Moisture content analysis of candies and chocolates of different brands available in the Bangladeshi market (each sample was assessed three times).

Brand code	Average M.C. (%)	Std. dev.
*Moisture content analysis of candies*		
S1	1.90	0.32
S2	1.56	0.078
S3	4.78	0.33
S4	2.22	0.21
S5	3.34	0.28
S6	4.34	0.064
S7	2.03	0.21
S8	2.57	0.078
S9	1.96	1.16
S10	2.63	0.30
S11	0.95	0.0495
S12	1.86	0.24
S13	4.04	0.29
S15	1.6	0.13
S16	1.08	0.29
S17	1.940	0.014
S18	1.05	0.02
S19	0.70	0.07
S20	0.64	0.03
S21	0.99	0.26
S22	1.02	0.04
S23	1.23	0.03

*Moisture content analysis of chocolates*		
S14	1.96	0.085
S27	1.04	0.042
S28	1.71	0.071
S29	0.88	0.031
S31	0.98	0.028

**Table 5:** Sulphated ash analysis of candies and chocolates of different brands available in the Bangladeshi market (each sample was assessed three times).

Brand code	Average sulphated ash (%)	Std. dev.
*Sulphated ash analysis of candies*		
S1	20.39	0.57
S2	21.83	2.52
S3	25.72	1.02
S4	21.54	1.11
S5	22.23	2.068
S6	22.70	1.98
S7	22.01	0.74
S8	22.32	0.198
S9	21.02	0.34
S10	22.42	0.89
S11	22.97	3.24
S12	22.93	1.55
S13	22.79	4.35
S15	20.93	1.44
S16	18.81	1.16
S17	20.99	2.26
S18	19.76	2.35
S19	19.22	1.21
S20	21.78	2.95
S21	22.26	0.81
S22	21.32	1.51
S23	23.061	0.18

*Sulphated ash analysis of chocolates*		
S14	20.71	0.68
S27	20.62	0.33
S28	20.19	0.46
S29	19.93	1.16
S30	21.19	0.64
S31	21.23	1.32

**Table 6:** Heavy metal analysis in different brands of candies and chocolates available in the Bangladeshi market. Each sample was assessed three times.

Brand code	Lead (*μ*g/g) ± STDEV	Chromium (*μ*g/g) ± STDEV	Nickel (*μ*g/g) ± STDEV	Cadmium (*μ*g/g) ± STDEV
*Heavy metal analysis in candies*				
S1	1.72 ± 0.02	BDL	13.24 ± 2.28	0.098 ± 0.01
S2	1.25 ± 0.10	BDL	1.48 ± 0.32	0.11 ± 0.10
S3	0.75 ± 0.12	BDL	3.1 ± 0.02	0.08 ± 0.03
S4	0.95 ± 0.03	BDL	0.43 ± 0.10	0.14 ± 0.04
S5	0.24 ± 0.02	BDL	0.38 ± 0.04	0.22 ± 0.12
S6	1.43 ± 0.11	BDL	1.43 ± 0.52	0.17 ± 0.22
S7	0.97 ± 0.16	BDL	0.61 ± 0.05	0.097 ± 0.08
S8	1.44 ± 0.04	BDL	48.10 ± 4.20	0.072 ± 0.05
S9	1.23 ± 0.02	BDL	0.83 ± 0.013	0.13 ± 0.11
S10	2.40 ± 0.01	BDL	14.78 ± 1.87	0.15 ± 0.04
S11	1.23 ± 0.08	BDL	1.28 ± 0.1	0.23 ± 0.14
S12	1.23 ± 0.01	BDL	0.79 ± 0.08	0.22 ± 0.07
S13	1.21 ± 0.09	BDL	0.77 ± 0.12	0.19 ± 0.02
S15	1.08 ± 0.02	BDL	0.93 ± 0.18	0.15 ± 0.07
S16	1.11 ± 0.02	BDL	1.19 ± 0.22	0.19 ± 0.13
S17	1.10 ± 0.11	BDL	0.81 ± 0.11	0.13 ± 0.02
S18	1.08 ± 0.14	BDL	2.89 ± 0.59	0.12 ± 0.06
S19	1.03 ± 0.06	BDL	1.1 ± 0.06	0.08 ± 0.02
S20	1.01 ± 0.10	BDL	0.83 ± 0.14	0.071 ± 0.08
S21	0.96 ± 0.15	BDL	0.62 ± 0.24	0.099 ± 0.10
S22	0.96 ± 0.04	BDL	0.42 ± 0.12	0.097 ± 0.03
S23	0.93 ± 0.02	BDL	1.25 ± 0.04	0.13 ± 0.10
S32	BDL	11.11 ± 2.02	BDL	0.42 ± 0.17
S33	BDL	10.73 ± 1.52	0.81 ± 0.13	0.41 ± 0.04
S34	BDL	12.11 ± 2.2	0.58 ± 0.10	0.39 ± 0.02
S38	BDL	12.35 ± 1.79	0.40 ± 0.06	0.44 ± 0.11

*Heavy metal analysis in chocolates*				
S14	1.18 ± 0.56	BDL	0.83 ± 0.17	0.19 ± 0.02
S27	0.91 ± 0.21	BDL	1.57 ± 0.11	0.17 ± 0.05
S28	0.86 ± 0.12	BDL	1.14 ± 0.05	0.20 ± 0.01
S30	0.91 ± 0.10	BDL	1.43 ± 0.07	0.25 ± 0.07
S31	1.02 ± 0.02	0.50 ± 0.17	1.09 ± 0.27	0.22 ± 0.03
S35	BDL	12.19 ± 1.67	1.11 ± 0.18	0.38 ± 0.04
S36	BDL	11.54 ± 2.06	1.32 ± 0.51	0.42 ± 0.02
S37	BDL	11.75 ± 2.23	BDL	0.38 ± 0.05
S39	BDL	12.79 ± 1.72	0.90 ± 0.05	0.36 ± 0.10

BDL = below detection limit.

**Table 7:** Heavy metal analysis in wrappers of different brands of candies and chocolates available in the Bangladeshi market. Each sample was assessed three times.

Package code (brand code)	Lead (*μ*g/g)	Chromium (*μ*g/g)	Nickel (*μ*g/g)	Cadmium (*μ*g/g)	Total (*μ*g/g)
*Heavy metal analysis in candy wrappers*					
P1 (S1)	2.24 ± .81	BDL	2.10 ± 0.52	6.30 ± 1.33	10.64
P2 (S2)	6.25 ± 1.11	BDL	11.25 ± 2.14	4.38 ± 0.82	21.88
P3 (S3)	124.78 ± 10.2	BDL	9.47 ± 1.86	2.15 ± 0.55	136.4^∗^
P4 (S4)	58.44 ± 3.21	BDL	15.58 ± 3.70	3.25 ± 1.10	77.27
P5 (S5)	4.31 ± 0.57	BDL	2.16 ± 0.37	2.57 ± 0.25	9.04
P6 (S6)	17.24 ± 1.26	BDL	4.31 ± 1.13	4.31 ± 1.05	25.86
P7 (S7)	5.24 ± 0.78	BDL	3.72 ± 1.92	1.86 ± 0.72	10.82
P8 (S8)	25.25 ± 1.17	BDL	23.99 ± 3.21	8.84 ± 1.82	58.08
P9 (S9)	2.39 ± 0.72	BDL	2.87 ± 0.63	1.68 ± 0.06	6.94
P10 (S10)	27.81 ± 2.2	BDL	28.12 ± 2.1	6.49 ± 0.62	62.42
P11 (S11)	133.41 ± 4.89	BDL	9.07 ± 0.92	2.36 ± 0.54	144.84^∗^
P12 (S12)	347.78 ± 24.10	BDL	6.09 ± 0.75	3.94 ± 0.39	357.81^∗^
P13 (S13)	512.5 ± 14.22	BDL	18.5 ± 1.62	5.5 ± 1.19	536.5^∗^
P15 (S15)	367.95 ± 5.52	BDL	35.76 ± 3.10	3.78 ± 0.52	407.49^∗^
P16 (S16)	761.94 ± 21.02	BDL	24.58 ± 2.22	7.72 ± 1.16	794.24^∗^
P17 (S17)	988.01 ± 11.32	BDL	34.13 ± 1.72	11.07 ± 2.28	1033.21^∗^
P18 (S18)	581.76 ± 19.70	BDL	24.11 ± 3.06	6.29 ± 1.43	612.16^∗^
P19 (S19)	638.27 ± 15.53	BDL	18.81 ± 1.86	7.19 ± 1.78	664.27^∗^
P20 (S20)	576.62 ± 21.09	BDL	25.05 ± 2.72	5.89 ± 0.77	607.56^∗^
P21 (S21)	321.51 ± 7.1	BDL	13.02 ± 1.55	3.33 ± 1.02	337.86^∗^
P22 (S22)	598.79 ± 12.92	BDL	14.11 ± 1.92	6.55 ± 0.59	619.45^∗^
P23 (S23)	460.87 ± 24.61	BDL	12.54 ± 0.49	4.94 ± 0.92	478.35^∗^
P32 (S32)	7.1 ± 1.4	1763.75 ± 18.02	9.72 ± 1.35	23.75 ± 3.70	1804.32^∗^
P33 (S33)	10.0 ± 1.42	1062.5 ± 15.52	6.40 ± 0.43	22.5 ± 4.21	1101.4^∗^
P34 (S34)	37.92 ± 3.27	450.83 ± 11	11.12 ± 1.09	5.42 ± 0.52	505.29^∗^
P38 (S38)	35.03 ± 5.18	1112.45 ± 23.05	7.80 ± 1.39	37.5 ± 3.75	1192.78^∗^

*Heavy metal analysis in chocolate wrappers*					
P14 (S14)	402.09 ± 20.33	BDL	6.14 ± 0.66	3.97 ± 0.63	412.2^∗^
P27 (S27)	687.5 ± 14.62	BDL	21.22 ± 2.63	8.03 ± 1.1	716.75^∗^
P28 (S28)	311.89 ± 9.83	BDL	23.82 ± 3.15	3.93 ± 0.28	339.64^∗^
P30 (S30)	2586.75 ± 50.39	BDL	108.05 ± 5.36	27.54 ± 2.51	2722.34^∗^
P31 (S31)	385.97 ± 5.60	2.49 ± 0.82	11.75 ± 1.13	4.02 ± 0.87	404.23^∗^
P36 (S36)	BDL	280.04 ± 4.29	9.60 ± 1.39	9.375 ± 1.60	299.015^∗^
P37 (S37)	BDL	328.33 ± 8.14	BDL	13.33 ± 2.02	341.66^∗^
P39 (S39)	BDL	1092.5 ± 21.73	16.70 ± 2.53	45.0 ± 3.36	1154.2^∗^

BDL = below detection limit. ^∗^Exceeds the maximum limit (100 *μ*g/g) established in the EU Directive (94/62/EC) [46].

**(a):** 

Microbiological count of candy and chocolate brands (log CFU/g)										
Microbiological parameters	S3		S4		S16		S20		S24	
	Before enrichment	After enrichment	Before enrichment	After enrichment	Before enrichment	After enrichment	Before enrichment	After enrichment	Before enrichment	After enrichment
Total aerobic bacterial count	3.48	NA	3.34	NA	<1.0	NA	<1.0	NA	<1.0	NA
Total coliform count	<1.0	Absent	<1.0	Absent	<1.0	Absent	<1.0	Absent	<1.0	Absent
*Escherichia coli*	<1.0	Absent	<1.0	Absent	<1.0	Absent	<1.0	Absent	<1.0	Absent
*Salmonella* spp.	<1.0	Absent	<1.0	Absent	<1.0	Absent	<1.0	Absent	<1.0	Absent
*Pseudomonas* spp.	<1.0	Absent	<1.0	Absent	<1.0	Absent	<1.0	Absent	<1.0	Absent
*Bacillus* spp.	2.60	Present	<1.0	Absent	<1.0	Present	<1.0	Absent	<1.0	Absent

**(b):** 

Microbiological count of candy and chocolate brands (log CFU/g)										
Microbiological parameters	S25		S26		S32		S33		S34	
	Before enrichment	After enrichment	Before enrichment	After enrichment	Before enrichment	After enrichment	Before enrichment	After enrichment	Before enrichment	After enrichment
Total aerobic bacterial count	<1.0	NA	<1.0	NA	<1.0	NA	<1.0	NA	<1.0	NA
Total coliform count	<1.0	Absent	<1.0	Absent	<1.0	Absent	<1.0	Absent	<1.0	Absent
*Escherichia coli*	<1.0	Absent	<1.0	Absent	<1.0	Absent	<1.0	Absent	<1.0	Absent
*Salmonella* spp.	<1.0	Absent	<1.0	Absent	<1.0	Absent	<1.0	Absent	<1.0	Absent
*Pseudomonas* spp.	<1.0	Absent	<1.0	Absent	<1.0	Absent	<1.0	Absent	<1.0	Absent
*Bacillus* spp.	<1.0	Absent	<1.0	Present	<1.0	Present	<1.0	Present	<1.0	Absent

**(c):** 

Microbiological count of candy and chocolate brands (log CFU/g)										
Microbiological parameters	S35		S36		S37		S38		S39	
	Before enrichment	After enrichment	Before enrichment	After enrichment	Before enrichment	After enrichment	Before enrichment	After enrichment	Before enrichment	After enrichment
Total aerobic bacterial count	3.41	NA	<1.0	NA	3.49	NA	<1.0	NA	<1.0	NA
Total coliform count	<1.0	Absent	<1.0	Absent	<1.0	Absent	<1.0	Absent	<1.0	Absent
*Escherichia coli*	<1.0	Absent	<1.0	Absent	<1.0	Absent	<1.0	Absent	<1.0	Absent
*Salmonella* spp.	<1.0	Absent	<1.0	Absent	<1.0	Absent	<1.0	Absent	<1.0	Absent
*Pseudomonas* spp.	<1.0	Absent	<1.0	Absent	<1.0	Absent	<1.0	Absent	<1.0	Absent
*Bacillus* spp.	<1.0	Present	<1.0	Absent	<1.0	Absent	<1.0	Present	<1.0	Present

NA = not applicable.

## Data Availability

Data is available upon request.
